# Zinc-mediated dynamics of CD4/CD8α co-receptors and Lck kinase: implications for zinc homeostasis, immune response, and biotechnological innovations

**DOI:** 10.1093/mtomcs/mfaf018

**Published:** 2025-06-12

**Authors:** Anna Kocyła, Artur Krężel

**Affiliations:** Department of Chemical Biology, Faculty of Biotechnology, University of Wroclaw, F. Joliot-Curie 14a 50-383 Wrocław, Poland; Department of Chemical Biology, Faculty of Biotechnology, University of Wroclaw, F. Joliot-Curie 14a 50-383 Wrocław, Poland

## Abstract

Zinc (Zn²⁺) plays a pivotal role in T-cell activation by modulating the interactions between the co-receptors CD4 and CD8α and the Src-family kinase Lck. A central structural feature in this regulation is the zinc clasp, a Zn²⁺-mediated CD4/CD8α-Lck receptor interface that stabilizes these complexes during T cell receptor signaling. Recent findings reveal that the stability of CD4-Lck and CD8α-Lck complexes is differentially regulated by Zn²⁺, which acts as a dynamic signaling molecule during T-cell activation. Here, we discuss the structural dynamics of these interactions and the impact of Zn²⁺ on CD4 dimerization, palmitoylation, and membrane interactions, which are crucial for effective T-cell responses. These mechanisms underscore a broader framework in which zinc biology intersects with co-receptor-Lck coupling to guide T-cell development, lineage fidelity, and functional specialization. Beyond immunobiology, zinc-dependent protein-protein interactions offer promising opportunities for biotechnological innovation, particularly in the design of molecular systems that exploit zinc-mediated structural control.

## Graphical Abstract

**Graphical Abstract:**
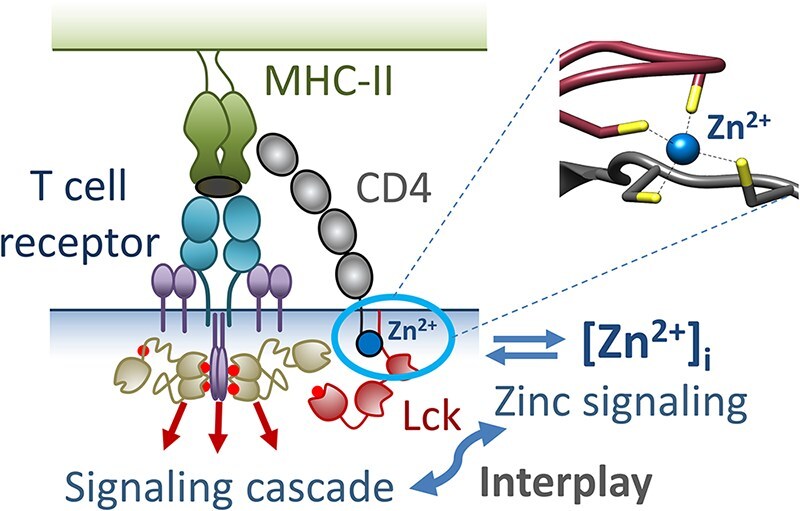

## Introduction

**Figure 1.**
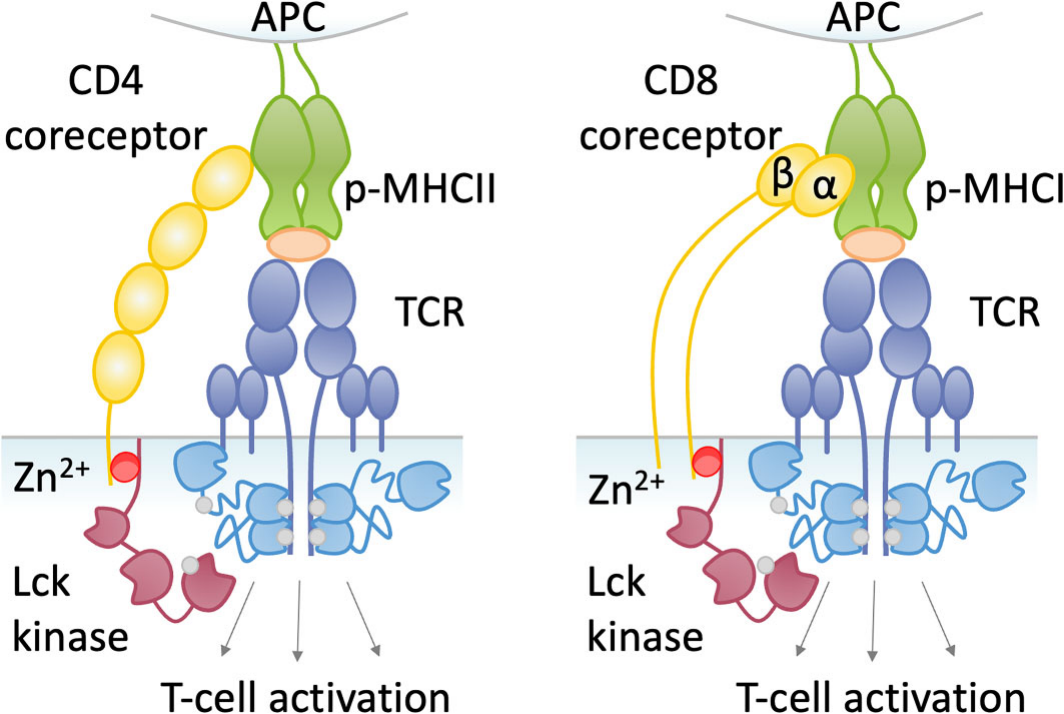
Antigen presentation via peptide-major histocompatibility complex class II (p-MHCII) and p-MHC class I complex that binds to the TCR of CD4^+^ and CD8^+^ cells, respectively. It allows its recognition by the T cell receptor, which is assisted by the CD4 coreceptor. After TCR-pMHCII-CD4 multiprotein complex assembly, intracellularly located lck tyrosine kinase associated with CD4/CD8α via Zn^2+^ phosphorylates TCR, enabling Zap70 recruitment, thus starting the signaling cascade.

The journey to understanding T-cell activation began in the late 1970s and early 1980s, a period marked by groundbreaking discoveries in immunology. Researchers focused on elucidating the complex mechanisms underlying the immune response, leading to the identification of various cell surface markers on T cells. In 1979, the discovery of the CD4 and CD8 co-receptors emerged as a pivotal moment in immunology [1]. CD4 was soon identified as a marker for helper T cells, while CD8 was recognized as a distinguishing feature of cytotoxic T cells—a differentiation that allowed scientists to categorize T cells by function [2]. As research progressed, the Src-family tyrosine kinase Lck was recognized as a critical player in T-cell signaling. By the mid-1980s, Lck was found to associate with both CD4 and CD8α co-receptors, acting as a key mediator of the signaling cascade initiated upon T cell receptor (TCR) engagement [3–5]. TCR signaling is initiated through the specific interaction of the TCR with an antigen-presenting major histocompatibility complex (MHC) molecule on the surface of an antigen-presenting cell, leading to the formation of a functional immunological synapse. The CD4 and CD8α co-receptors further stabilize this interaction by binding to MHC class II and MHC class I, respectively (Fig. 1). The discovery of CD4/CD8α-Lck interactions illuminated the intricacy of T-cell activation but also raised new questions: How stable are these interactions? Do CD4 and CD8α bind Lck in the same way, and could differences in binding affect the functional specialization of helper and cytotoxic T cells?

Subsequent work revealed that the interactions were indeed distinct. CD4 was found to bind Lck in a near-stoichiometric and stable fashion, while CD8α-Lck coupling was more transient and less complete [6, 7]. This suggested that CD4-Lck complexes function as stable signaling platforms during T-cell development and help maintain lineage stability, whereas CD8α-Lck complexes might allow for greater responsiveness to variable antigenic stimuli, reflecting the rapid action required of cytotoxic T cells. A comprehensive review by Davis and van der Merwe further elaborated on these findings, integrating existing research and providing a broader understanding of how the differential stability of CD4-Lck and CD8α-Lck complexes influences the functional specialization of T cell subsets and the mechanisms governing T-cell activation [8].

A key structural insight soon followed: the stable interaction between Lck and the co-receptors is mediated by a zinc ion (Zn^2+^), which bridges conserved cysteine residues in the membrane-proximal domains of both Lck and CD4 or CD8α [9, 10]. It was shown by NMR analysis that co-receptor tails and the Lck N-terminus are unstructured in isolation but assemble with Zn^2+^ to form compactly folded “zinc clasp” cores that are augmented by distinct structural elements [11]. For many years, Zn^2+^-mediated interaction was regarded as purely structural and static—an elegant molecular clasp that ensured stable coupling. However, more recent research on zinc homeostasis in immune cells has begun to challenge that static view. Intracellular Zn^2+^ concentrations are known to fluctuate during T-cell activation, regulated by zinc transporters [12]. These dynamic changes raise the intriguing but largely unexplored possibility that changes in intracellular Zn^2+^ concentrations could influence the assembly or stability of CD4-Lck and CD8α-Lck complexes in real time, adding an additional regulatory layer to T-cell signaling that has yet to be fully investigated.

This article explores the interplay between co-receptor—Lck coupling and zinc biology and biochemistry, moving beyond static structural descriptions toward a dynamic understanding of how metal ion homeostasis may fine-tune T-cell signaling. We begin with a short overview of the regulation of Zn^2+^ in immune cells and how fluctuations in intracellular Zn^2+^ concentrations intersect with key signaling pathways. Building on this, we explore the structural and biophysical principles underlying the Zn^2+^-mediated stabilization of CD4-Lck and CD8α-Lck complexes. Particular attention is given to the dynamic properties of CD4 itself—including its dimerization, palmitoylation, and membrane compartmentalization—and how these processes may be modulated by Zn^2+^ availability. We then consider the functional consequences of these molecular mechanisms in T-cell development, lineage fidelity, and effector function. Finally, we look outward, discussing how the zinc clasp domain has been harnessed in biotechnology and how Zn^2+^-dependent protein-protein interactions may represent a broader regulatory principle across biological systems.

## Zinc homeostasis in a T cell

### Zinc signaling and transport mechanisms in T cells

Zinc is a nutritionally essential trace mineral required for more than 10% of encoded proteins to function properly. The human body contains 2–3 g of zinc depending on factors such as age, sex, and overall health [13]. It is primarily stored in the muscles and bones, but it is also present in blood with 80–85 µg/dL levels in blood serum, where it plays a vital role in various biochemical processes [14]. In blood plasma, about 75%–85% of zinc is bound to albumin, with the rest complexed to α2-macroglobulin and other proteins [15]. Only a small fraction exists as free Zn²⁺, the bioactive form that is tightly regulated under physiological conditions [16]. Changes in serum labile Zn^2+^, particularly during stress, infection, or inflammation, have profound effects on systemic Zn^2+^ distribution and subsequently influence cellular Zn^2+^ availability. During inflammatory responses, elevated levels of proinflammatory cytokines such as IL-6 and TNF-α, have been correlated with increasing percentages of subjects with low circulating Zn^2+^ concentrations [17]. Among several contributing mechanisms, these cytokines have been shown to stimulate hepatic synthesis of metallothioneins (MTs) and acute-phase proteins, which may facilitate Zn^2+^ sequestration in the liver and contribute to decreased serum labile Zn^2+^ levels [18–20]. Importantly, this process reflects a regulated redistribution of Zn^2+^ rather than systemic deficiency and constitutes part of the broader acute phase response. A contributing factor to declining serum Zn^2+^ is the reduced synthesis of albumin during inflammation, which diminishes the zinc buffering capacity of plasma. Furthermore, recent evidence shows that elevated levels of nonesterified free fatty acids, common during metabolic stress, can allosterically impair albumin's Zn^2+^ binding site, resulting in the release of Zn²⁺ and redistribution to alternative plasma proteins such as histidine-rich glycoprotein and complement system components [21]. These mechanisms alter not just total Zn^2+^ content, but also Zn^2+^ speciation, influencing its cellular uptake and signaling roles. At the cellular level, changes in extracellular zinc are sensed through zinc transporters and membrane-associated zinc sensing mechanisms. Members of the ZIP (SLC39A) transporter family respond to low extracellular Zn^2+^ by increasing cellular influx, while ZnT (SLC30A) transporters mediate efflux or organellar sequestration to maintain homeostasis (see below). Additionally, certain cells express GPR39, a G-protein-coupled receptor that is activated by extracellular Zn²⁺. GPR39 engagement leads to nontranscriptional signaling cascades that influence both transporter expression and other cell-specific responses to zinc availability [22]. This receptor-based zinc sensing highlights a key mechanism by which systemic Zn^2+^ redistribution is translated into cellular physiological adaptations.

**Figure 2.**
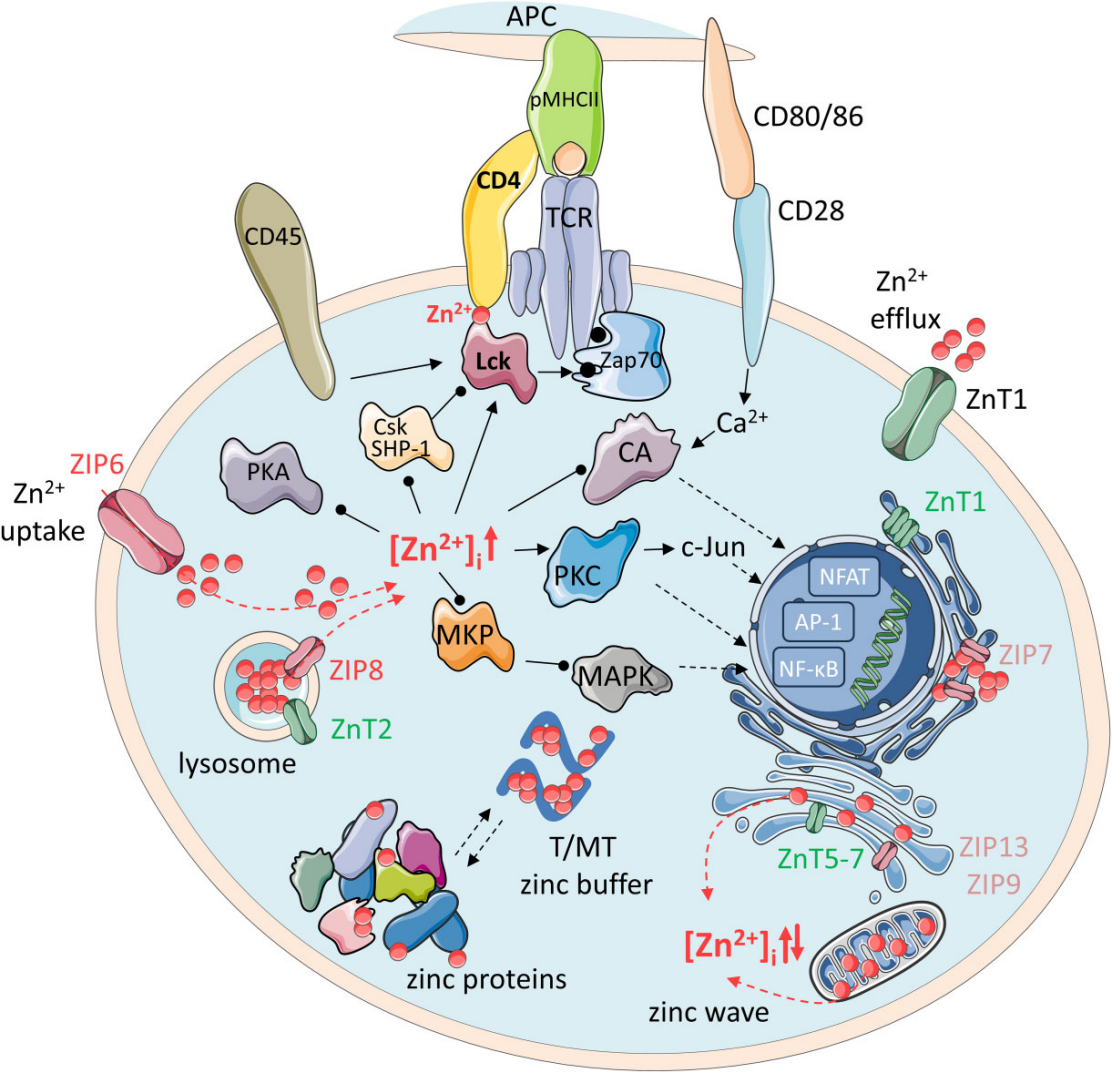
Schematic representation of cellular zinc homeostasis. ZIP and ZnT are zinc transporters responsible for Zn^2+^ uptake and efflux systems, respectively. In the cell, Zn^2+^ is shuttled between zinc proteins, MT/T (metallothionein/thionein) buffer, and Zn^2+^ stores. In the case of the TCR activation, surplus Zn^2+^ appears in the cell. Zn^2+^ is released from the intracellular stores, increasing intracellular labile Zn^2+^ concentration ([Zn^2+^]_i_). Therefore, multiple proteins in the T-cell signaling pathways are affected. The straight-line arrow indicates activation, the straight line with a dot at the end represents inhibition. Abbreviations: APC, antigen-presenting cell; pMHCII, peptide-major histocompatibility complex Class II; TCR, T cell receptor; PKA, protein kinase A; Csk, C-terminal Src kinase; SHP-1, Src homology region 2 domain-containing phosphatase-1; Zap70, zeta-chain-associated protein kinase 70; CA, calmodulin-dependent protein kinase; PKC, protein kinase C; MKP, mitogen-activated protein kinase phosphatase; MAPK, mitogen-activated protein kinase; NFAT, nuclear factor of activated T-cells; AP-1, activator protein 1; NF-κB, nuclear factor kappa-light-chain-enhancer of activated B cells.

Zn^2+^ in the cell is distributed to its target sites, which are mainly proteins in which Zn^2+^ plays a catalytic or structural role. In both of these types of proteins, Zn^2+^ is a persistent cofactor, without which the function of these proteins is not performed. This is accomplished mainly through a thermodynamic and kinetic pathway, according to which Zn^2+^ is associated with a high stability constant that prevents Zn^2+^ dissociation under cellular conditions [23, 24]. The low *k*_off_ kinetic constant particularly typical for enzymes further impedes this dissociation in cellular time windows. These proteins are a static reservoir of Zn^2+^, however, their degradation or damage may eventually lead to the release of the metal. Nevertheless, before Zn^2+^ binds to the static target proteins, it is present in cystosol in the so-called mobile form also known as labile. The most important components of the dynamic zinc pool are MTs (the four main MT1-MT4 isoforms), small polycysteine proteins that bind up to seven Zn^2+^ ions with varying affinities from nano- to picomolar and play zinc buffering roles together with their apo-forms (thioneins, T) forming Zn^2+^-depleted species (Zn_7-x_MTs) (Fig. 2) [25, 26]. Apart from MTs, other proteins or low-molecular-weight ligands, such as glutathione, amino acids, carboxylic acids, and anions, serve as transient zinc ligands and stores and form the basis of mobile Zn^2+^ [27]. It is important to note that the cellular zinc metalloproteome is not composed solely of static proteins [28]. A significant number of proteins exhibit dynamic characteristics, such as those that are transiently modulated or regulated by Zn²⁺. The activity of these proteins depends on fluctuations in labile Zn²⁺ levels. An increase in local Zn²⁺ concentration leads to Zn²⁺ binding, resulting in protein activation, inhibition, or assembly. For instance, this assembly occurs in interprotein complexes, where Zn²⁺ binds to two or more protomers [29]. Conversely, a decrease in available Zn²⁺ results in metal dissociation, allowing proteins to return to their resting state before activation. Such dynamic Zn²⁺ binding sites are characterized by moderate Zn^2+^ affinity and are found in a wide variety of proteins, including some enzymes, zinc finger-like motifs, and allosteric sites [29–31].

Beyond static zinc buffering, its regulation in cells follows a more complex and dynamic model that accounts for time-dependent shifts in cytosolic Zn^2+^ concentrations [32]. This model incorporates mechanisms such as Zn^2+^ influx, efflux, and sequestration across compartments, reflecting the utility of Zn^2+^ as a signaling molecule. Transient changes in intracellular Zn^2+^ are thus increasingly recognized for their role in signal transduction, protein activity modulation, and subcellular re-localization.

Two major transporter families maintain intracellular Zn^2+^ re-distribution: ZIP (Zrt-, Irt-like protein) importers and ZnT (zinc transporter) exporters (Fig. 2). ZIPs are primarily responsible for the influx of Zn^2+^ into the cytosol, whereas ZnT exporters facilitate the movement of Zn^2+^ out of the cytosol, maintaining cellular homeostasis [33]. Among the 14 ZIP family members, ZIP6, ZIP8, and ZIP13 are highly expressed in human T cells. Upon TCR stimulation, ZIP6 was found predominantly localized to lipid rafts in the immune synapse, where its phosphorylation increases within minutes due to interaction with the key early T-cell signaling kinase, Zap70 [34]. ZIP6’s role in zinc influx is not limited to early signaling but extends to transcriptional activity associated with Zn^2+^ influx, expression of MTs, and promotion of T-cell proliferation, survival, and expansion [35]. ZIP8 and ZIP13, on the other hand, are primarily expressed on the lysosome and ER/Golgi membranes in T cells. ZIP8 facilitates the transport of Zn^2+^ from the lysosome to the cytoplasm upon T-cell stimulation, promoting the release of pro-inflammatory cytokines like IFN-γ [36]. The role of ZIP13 remains less explored but may involve the modulation of cellular stress response due to its unique localization in the ER/Golgi [36, 37].

TCR engagement triggers a rapid and transient increase in intracellular Zn^2+^ concentrations through ZIP6 and ZIP8 at the early stage of signaling [38]. This influx enhances T-cell signaling by inhibiting phosphatases such as SHP-1 and potentiating kinases including Zap70, LAT, and Lck [34, 35, 39]. In contrast, long-term zinc signals contribute to sustained transcriptional responses and T-cell proliferation. ZIP8, as a transcriptional target of NF-κB, links Zn^2+^ to inflammatory responses via mTORC1 [40, 41]. Similarly, ZIP6 supports long-term activation and T-cell expansion, reinforcing the dual role of Zn^2+^ in immune regulation [35].

### Zinc-dependent regulation of TCR signaling pathways

Lck plays a pivotal role in initiating TCR signaling by phosphorylating all 10 immunoreceptor tyrosine-based activation motifs (ITAMs) located in the CD3ζ and CD3ε subunits of the TCR complex, thereby facilitating the recruitment and activation of downstream kinases, such as Zap70 (Fig. 2). The coreceptors CD4 and CD8 guide Lck to the immunological synapse, further amplifying signaling. Zn^2+^ influx, particularly via ZIP6, plays a critical role in modulating this process. One important function of Zn^2+^ is the inhibition of SHP-1, a phosphatase known to negatively regulate Lck activity by dephosphorylating its activatory tyrosine (Y394). By inhibiting SHP-1, Zn^2+^ influx sustains the active state of Lck, thereby enhancing signal propagation during the early phase of T-cell activation [38]. Furthermore, Lck regulation is finely tuned through structural mechanisms, including its capacity to form homodimers and through dual-site phosphorylation: activatory tyrosine (Y394) and inhibitory tyrosine (Y505) [42, 43]. The inhibitory Y505 site is phosphorylated by C-terminal Src kinase (Csk), rendering Lck inactive in resting cells. Upon TCR engagement, this inhibitory phosphate is removed by CD45, a membrane-associated phosphatase, which enables Lck to undergo auto- and trans-phosphorylation at Y394, transitioning it into an active conformation. Interestingly, the open, active form of Lck remains subject to negative regulation via dephosphorylation of Y394 by both SHP-1 and CD45, underscoring the dynamic balance of kinase and phosphatase activities.

Intracellular zinc signals intersect with this regulatory network at multiple levels. Beyond inhibiting SHP-1, Zn^2+^ has also been shown to suppress the activity of other protein tyrosine phosphatases (PTPs), including Csk, thereby promoting prolonged Lck activation [39, 44, 45]. Notably, inhibition of Csk prevents re-phosphorylation of the inhibitory Y505 site, allowing Lck to remain active [46]. Furthermore, the regulation of Csk itself involves cAMP-dependent protein kinase A (PKA): Csk is re-activated via phosphorylation by PKA, and PKA activity depends on intracellular cAMP levels. Zn^2+^ homeostasis indirectly affects this pathway by modulating cAMP synthesis, thereby offering an additional layer of control over Csk and, consequently, Lck function [47–49].

Zn^2+^ signaling in T cells operates on two distinct temporal levels: rapid influxes and long-term transcriptional regulation. Upon TCR engagement, a fast Zn^2+^ influx, primarily mediated by transporters ZIP6 and ZIP8, plays a critical role in initiating downstream pathways such as NF-κB and MAPK. These transient signals support early T-cell activation, enhancing cytokine production and effector responses [50]. Although a slower Ca^2+^-dependent “zinc wave” has been reported in mast cells, T cells rely heavily on rapid Zn^2+^ influx [51]. Among the zinc-sensitive transcriptional regulators, NFAT (nuclear factor of activated T cells) is particularly notable. Following CD28 co-stimulation, Ca^2+^ influx activates calcineurin, a Ca^2+^/calmodulin-dependent phosphatase, which dephosphorylates NFAT to enable its nuclear entry [52–54]. Zn^2+^ regulates calcineurin both structurally and functionally, with inhibitory effects at certain concentrations, highlighting its dual role in modulating NFAT signaling.

Parallel to NFAT regulation, Zn^2+^ also regulates the activity of the protein kinase C (PKC) family. PKC isoforms are zinc metalloenzymes that bind up to four Zn^2+^ ions and are essential for T-cell activation, proliferation, and cytokine production [55–57]. Late-phase zinc signals regulate PKC activation, while PKC itself can alter intracellular Zn^2+^ by releasing it during oxidative thiol modifications, creating a feedback loop between Zn^2+^ and kinase activity [58–60]. Long-term zinc signaling extends beyond PKC to transcriptionally controlled zinc transporters such as ZIP8, which is induced by NF-κB during inflammation and regulates IL-1β production through the MTORC1 pathway [40, 41]. ZIP6, similarly, influences long-term T-cell function by regulating MT expression, promoting survival, and proliferation [35]. Furthermore, Zn^2+^ enhances IL-2-dependent T-cell proliferation, reinforcing its central role in sustained immune activation [61].

Beyond activation, Zn^2+^ is vital for T-cell differentiation, especially for Th17 cells. A physiological Zn^2+^ flux is required for optimal Th17 differentiation, as shown by impaired TCR signaling (via NF-κB and MAPK) upon ZIP8 downregulation [62]. Zn^2+^ can also function as a costimulatory signal in the absence of CD28, supporting T helper cell polarization [63]. However, dysregulated Zn^2+^ influx—either excessive or deficient—may impair these critical pathways, compromising immune outcomes. In summary, Zn^2+^ shapes both the initiation and duration of T-cell responses: short-term signals amplify immediate activation, while long-term effects influence transcriptional activation, differentiation, and survival.

## Biophysical and structural aspects of zinc-mediated CD4/CD8α-Lck assemblies

**Figure 3.**
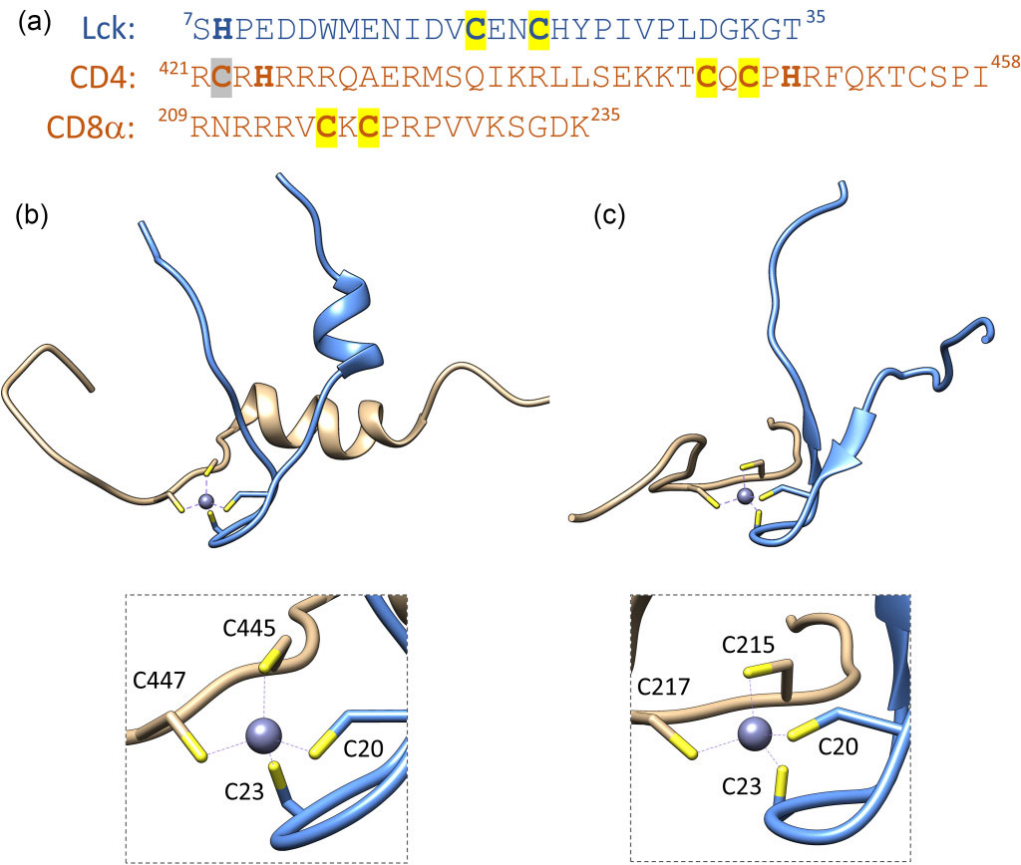
NMR structures of zinc clasp domains. (a) Amino acid sequences of peptides were used for structural investigations. Bold highlights Cys and His residues, while yellow and gray colors indicate Zn²⁺ coordinating and palmitoylated cysteine residues, respectively. Structural representation of (a) Zn(CD4)(Lck) and (c) Zn(CD8)(Lck) complexes with highlighted cysteine residues that coordinate Zn²⁺ intermolecularly.

Based on previous structure-function investigations and initial NMR assessments of the full-length cytoplasmic tails of CD4 and CD8α co-receptors and the unique region of Lck, Eck and colleagues proposed specific short fragments of the interacting molecules [6, 11, 64]. For the human CD4 co-receptor, the peptide included residues from 421 to 458, representing the entire cytosolic tail. In contrast, the CD8α fragment consisted of residues from 209 to 235 and notably omitted the last eight residues of the cytoplasmic region. For Lck, the selected fragment encompassed residues from 7 to 35 (Fig. 3a). The structures of the Zn(CD4)(Lck) and Zn(CD8α)(Lck) complexes were determined using NMR and are shown in Fig. 3a and c, respectively. The CD4 complex reveals a short α-helix at the N-terminus of the CD4 tail, alongside an additional α-helix at the N-terminus of Lck. These amphipathic helices are arranged roughly perpendicularly, creating a compact hydrophobic core. Following the N-terminal helix in Lck, a β-hairpin structure positions two cysteine residues for effective metal coordination at its apex. The C-terminal segment of the CD4 tail loops over the β-hairpin, with its CxCP motif facilitating the tetrahedral coordination of the bound Zn^2+^ ion. Beyond the CxCP motif, the CD4 tail is primarily unstructured. The interface between CD4 and Lck is predominantly hydrophobic, except for the metal coordination sphere, and the predominance of basic and acidic residues in CD4 and Lck, respectively, does not contribute significantly to polar interactions. The CD8α complex exhibits a structural resemblance to the Zn^2+^-coordinating core observed in the CD4 complex (Fig. 3c). In the presence of CD8α, residues Asp18 to Ile27 of Lck form a zinc hairpin structure analogous to that seen in the CD4 complex, while the CxCP motif in CD8α adopts a conformation that can be superimposed with that in CD4. The N-terminal helices present in the CD4 complex are absent in the CD8α complex, which is expected due to the shorter length of the CD8α tail that contains only one hydrophobic residue preceding the CXCP motif. Consequently, it cannot form an amphipathic helix or any complementary hydrophobic surface, thus failing to promote the formation of the N-terminal helix in Lck. In addition to Zn^2+^ coordination, there appear to be relatively few intermolecular interactions that contribute to the stabilization of the complex, with the interface between Lck and CD8α being significantly smaller than that in the CD4 complex.

Peptide interaction studies were initially conducted using isothermal titration calorimetry (ITC), a method frequently employed to investigate protein-protein and protein-ligand interactions [11]. Experiments performed at pH 7.0 yielded dissociation constants (*K*_d_ = [CD4]⋅[Lck]/[Zn(CD4)(Lck)] or [CD8α]⋅[Lck]/[Zn(CD8α)(Lck)] of 0.4 µM for the CD4-Lck interaction and 0.9 µM for the CD8α-Lck interaction. It is important to note that this investigation did not account for Zn^2+^ as a reagent; although it was present under the experimental conditions, it was not included in the calculations. Additionally, the stoichiometry of the interactions was found to deviate from the typical 1:1 ratio expected for protein-protein complexes. Consequently, the *K*_d_ values obtained through ITC may underestimate the actual affinity, although they did indicate that the shorter CD8α form interacts significantly more weakly with Lck compared to CD4. A similar discrepancy between the coreceptor fragments was observed in spectroscopic studies using Co(II) as a spectroscopic probe for Zn^2+^ ions [65]. The complex formation (binding) constant (*K*_12_ = [Zn(CD4)(Lck)]/[Zn^2+^]_free_⋅[CD4]⋅[Lck] that accurately describes the affinity of Lck for CD4 in the presence of Zn^2+^ was determined through competition with the chromogenic chelator PAR (4-(2-pyridylazo)resorcinol) [66]. The term “12” in the low index refers to the two-step process in which Zn(CD4)(Lck) heterocomplex is formed. The *K*_12_ derived from this competition was found to be 5 × 10^18^ M^−2^ (at pH 7.4), indicating a significant increase compared to the direct titration results from ITC. To achieve a more accurate determination of the *K*_12_ constant, the peptides were fluorescently labeled, either with the fluorescent proteins Clover and mRuby2 or TAMRA, allowing for precise studies of hetero-FRET while selectively minimizing the effects of homodimerization [66]. The peptides were equilibrated in a series of partially Zn^2+^-saturated common chelators to obtain a wide range of free Zn^2+^ concentrations (pZn, −log[Zn^2+^]_free_) from 8 to 15. This indicated that the Zn(CD4)(Lck) complex was formed with a *K*_12_ = 4⋅10^18^ M^−2^ at pH 7.4. Determining this heterocomplex constant formation was critical for further analysis of complex formation under cellular variations in labile Zn^2+^ concentration (see below). However, it should be noted that there is a constant formation of free chemical species, and all of these should be considered in any calculations or simulations. Currently, no stability constant for Zn(CD8α)(Lck) has been established, but it is anticipated to be lower than that of Zn(CD4)(Lck) due to a lower number of stabilizing interactions within the domain (see above).

## Zinc-dependent regulation of CD4: structural dynamics and membrane interactions in T-cell signaling

Understanding the intricate dynamics of CD4 is crucial, as it plays a significant role in the functional modulation of T-cell signaling and the immune response. In this chapter, we will explore how zinc-dependent mechanisms influence these dynamics, particularly CD4 dimerization, palmitoylation, and membrane partitioning—shedding light on the molecular interactions that govern CD4’s behavior within the cellular membrane environment. This exploration enhances our comprehension of CD4’s role in immunology and opens avenues for potential therapeutic interventions targeting CD4-related pathways.

### Dynamic equilibrium of zinc-mediated CD4 homodimers and CD4-Lck heterodimers

**Figure 4.**
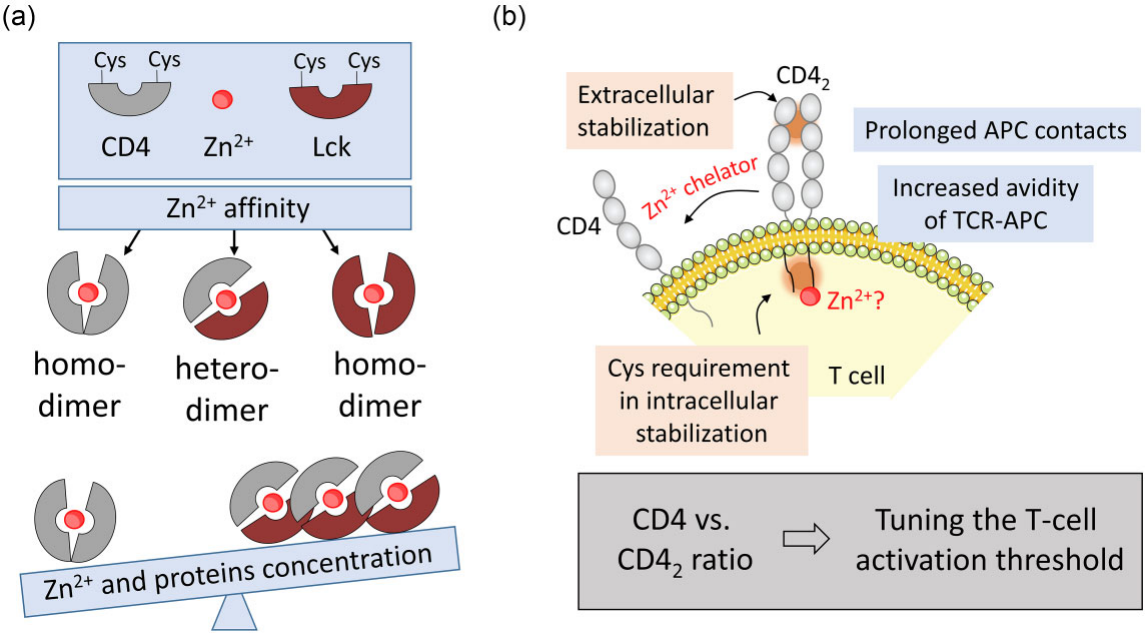
(a) Depiction of different complexes formed by Zn^2+^, CD4, and Lck that are characterized by homo- or heterodimeric stoichiometry. Zn^2+^ affinity and concentration of complex constituents dictates which complex forms preferentially. (b) Dimerization of CD4 coreceptor on a T-cell surface that affects the threshold needed for T-cell activation. The (CD4)_2_ complex is stabilized extra- and intracellularly with the impact of cysteine residues in the CD4 cytoplasmic tail. Abbreviations: APC, antigen-presenting cell; TCR, T cell receptor. Fig. 5b has been prepared based on data gathered in [67, 70].

**Figure 5.**
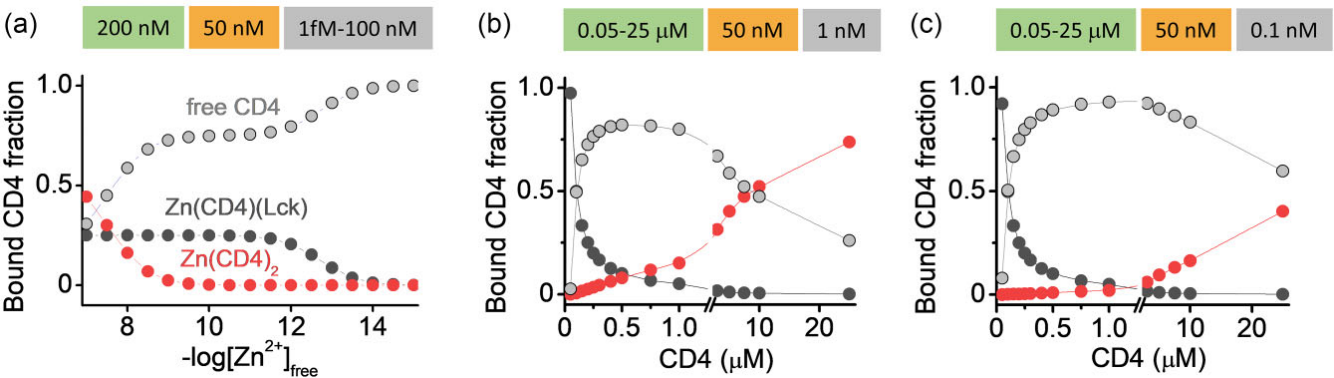
Equilibrium simulations were performed based on the stability constants determined for Zn²⁺ complexes with peptide models, which are represented as fractions of CD4. The concentrations of CD4, Lck, and free Zn²⁺ are depicted in green, orange, and gray boxes, respectively. (a) The species distribution of CD4 is shown as a function of −log[Zn²⁺]_free_. The total concentrations of CD4 and Lck were maintained at constant levels of 200 nM and 50 nM, respectively. The species distribution of CD4 was also analyzed as a function of the molar ratios of CD4 to Lck for free Zn²⁺ concentrations of 1 nM (b) and 0.1 nM (c) [71].

One of the key structural features of CD4 is its ability to form dimers, which enhance its function as a co-receptor in T-cell signaling (Fig. 4) [67, 68]. CD4 dimerization plays a crucial role in T-cell activation by stabilizing the interaction between the TCR and MHC-II molecules. Structural studies have demonstrated that CD4 dimerization increases the avidity of CD4 binding to MHC-II, thereby stabilizing the TCR—MHC-II interaction and enhancing the robustness of initial TCR engagement [69]. Biochemical evidence confirms the presence of CD4 dimers in various cell types, including primary T cells, highlighting their functional significance. Specific residues within the fourth extracellular domain, particularly K318 and Q344, are essential for dimer formation; mutations at these sites disrupt dimerization and impair CD4’s signaling capacity [67]. Beyond the extracellular domain, an additional dimerization interface is found within the cytoplasmic region, involving two cysteine residues [70]. The same cysteine residues coordinate Zn^2+^ in the CD4-Lck complex, however, only recent advances revealed that the dimerization is driven by Zn^2+^-dependent mechanisms (Fig. 4) [71]. Interestingly, these coordination residues contribute to both homodimer Zn(CD4)_2_ and heterodimer Zn(CD4)(Lck) formation, meaning that the availability of particular sequential elements leads to differences in stability and selectivity, as shown by minimal Zn^2+^-binding peptide models [65]. Elongation of the CD4 and Lck to those originally studied results in a marked preference for the heterocomplex, with its formation constant (*K*_12_) being four orders of magnitude higher than that of Zn(CD4)_2_ (*K*_12_ = 1.2 × 10^14^ M^−2^) [11, 51]. In T cells, both Zn(CD4)_2_ and Zn(CD4)(Lck) coexist, albeit at different intracellular labile Zn^2+^ concentrations, due to variations in their respective stability [71]. The differential affinity of CD4 homodimers and CD4—Lck for Zn^2+^ positions it as a dynamic regulator that modulates the balance between these two states. Stability simulations indicate that fluctuations in free Zn^2+^ concentrations significantly impact CD4 complex formation (Fig. 5). Specifically, increases in free Zn^2+^ from 0.1 to 10 nM promote Zn(CD4)_2_ formation, a finding supported by studies showing that CD4 responds to intracellular labile Zn^2+^ concentrations ranging from 0.1 to 0.6 nM in CD4^+^ Jurkat T cells [71]. While higher free Zn^2+^ concentrations have not yet been directly measured, localized fluctuations may occur [38]. Interestingly, the Zn(CD4)(Lck) proportion remains relatively stable at around 25% within physiologically relevant Zn^2+^ ranges, suggesting a finely tuned regulatory mechanism. The concentration of CD4 and Lck also influences complex formation. When CD4 and Lck levels increase at a constant ratio, Zn(CD4)_2_ formation is enhanced, whereas Zn(CD4)(Lck) levels remain stable. Determining the precise intracellular concentrations of these proteins and their associated Zn^2+^ complexes remains a challenge. However, it is known that Zn(CD4)(Lck) is locally enriched at the immunological synapse, emphasizing its potential role in T-cell activation.

Collectively, these findings highlight the critical role of Zn^2+^ in stabilizing the dimeric structure of CD4, which is essential for effective T-cell signaling and immune responsiveness. While CD4 dimerization is crucial for T-cell activation, excessive or unregulated dimerization may lead to dysregulated immune responses. This underscores the importance of maintaining a balanced monomer/dimer ratio. Understanding the regulatory mechanisms controlling this balance, including Zn^2+^ dynamics and membrane partitioning, clarifies CD4’s role in immune signaling and opens new avenues for therapeutic interventions in immune-related disorders. Rather than being a static structural event, CD4 dimerization emerges as a dynamic process that optimizes T-cell activation and signaling efficiency.

### CD4 palmitoylation and membrane partitioning: implications for T-cell signaling

CD4 palmitoylation is a critical post-translational modification that influences the localization and functionality of CD4 within the cellular membrane. This process involves the reversible addition of palmitic acid, a saturated fatty acid, to specific cysteine residues on CD4 (Cys419 and Cys422), CD8α (Cys206), and Lck, enhancing their association with lipid rafts and membrane microdomains [72, 73]. By modulating CD4’s membrane partitioning, palmitoylation plays a vital role in optimizing TCR signaling and the overall immune response [74]. The palmitoylation of CD4 has been shown to regulate the clustering of signaling molecules within lipid rafts, which is essential for the formation of the immunological synapse. This complex structure facilitates the spatial organization of critical signaling molecules such as Lck and PKC, thereby enhancing TCR signaling upon antigen recognition [72]. Studies have demonstrated that disruption of CD4 palmitoylation impairs its localization in lipid rafts, leading to defective TCR signaling and activation [74]. Furthermore, failure of CD4 palmitoylation can induce T-cell anergy, underscoring its crucial role in maintaining proper T-cell activation and immune response [75].

Recent studies have provided insights into the biochemical effects of CD4 palmitoylation, revealing that the incorporation of a long fatty acid chain in the CD4 cytoplasmic tail significantly alters its Zn^2+^ affinity. In solution, palmitoylated CD4 exhibited a Zn^2+^ affinity reduced by one order of magnitude, and when incorporated into model membranes, CD4-Lck interaction was completely abolished [71]. While results from model membranes may not directly translate to live cell conditions due to the lack of spatiotemporal membrane organization, these findings suggest that palmitoylation significantly affects CD4 flexibility and its interactions with other proteins. Understanding the dynamics of CD4 palmitoylation is essential for unraveling its contributions to T-cell activation and exploring potential therapeutic strategies targeting immune-related disorders [76, 77].

**Figure 6.**
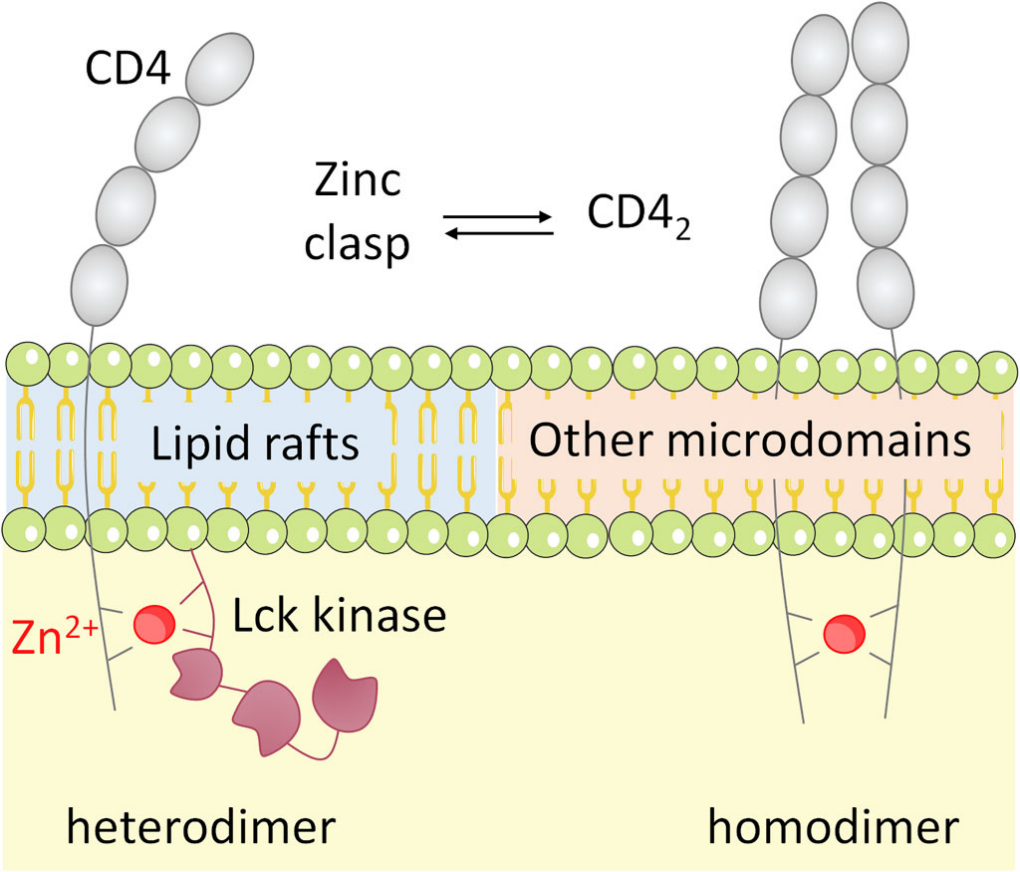
Depiction of CD4 dimerization in the plasma membrane. Whereas zinc clasp resides mainly in lipid rafts, dimeric CD4 has been found in distinct microdomains. The figure has been prepared based on data gathered in [70].

Beyond palmitoylation, CD4’s membrane localization and function are also influenced by the overall lipid composition of the membrane and its partitioning into specific microdomains. It has been proposed that two distinct pools of CD4 coexist in the plasma membrane, potentially influencing its pathophysiological activity (Fig. 6) [70, 73]. Investigations into CD4 distribution have indicated that the zinc clasp domain is predominantly found in lipid rafts, while CD4 homodimers are localized in tetraspanin-enriched microdomains. The spatiotemporal regulation of signaling events by lipid-based platforms has been extensively studied, particularly concerning immunological synapse formation [78, 89]. The organization of T-cell signaling molecules on the membrane follows a complex hierarchical structure. In nonactivated T cells, CD4, TCR, and Lck are clustered in small protein islands. Upon activation, these microclusters redistribute into supramolecular activation clusters with enhanced segregation, a process thought to limit random interactions and optimize signaling efficiency [79, 80]. The formation of discrete receptor and signaling molecule microdomains upon T-cell activation is believed to be driven by protein-protein interactions [81]. Identified Lck/CD2/LAT co-clusters can trap or exclude signaling proteins through diffusional trapping, thereby facilitating signaling by increasing local protein concentration.

The enrichment of CD4 in lipid rafts is dependent on both its palmitoylation and its association with Lck [72]. This localization is crucial for CD4 aggregation, which correlates with its ability to phosphorylate TCR and initiate downstream signaling. While CD4 has been extensively studied in terms of membrane organization, Lck, a cytoplasmic kinase, also plays a significant role in membrane dynamics. Lck is anchored to the membrane via myristoylation of its N-terminus, a modification that affects the formation of the zinc clasp complex. However, in resting cells, Lck is primarily freely diffusing [82, 83]. In summary, CD4 palmitoylation is a key regulatory mechanism governing its membrane localization, association with lipid rafts, and interaction with critical signaling molecules such as Lck. This modification, in conjunction with lipid microdomain distribution and protein-protein interactions, orchestrates TCR signaling and immune activation. A deeper understanding of these mechanisms may provide novel insights for therapeutic interventions targeting immune-related diseases.

## Zinc clasp assembly: from *in vitro* mechanisms to cellular context

**Figure 7.**
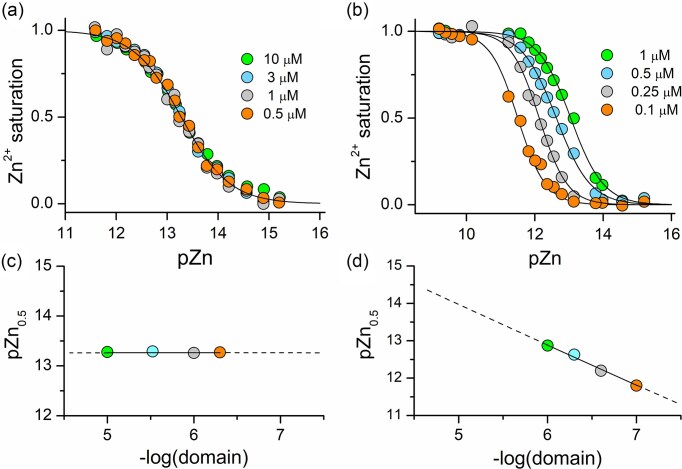
Correlation between the molar fraction of functional zinc domain (saturated with Zn²⁺) and free/labile Zn^2+^ concentration (pZn = −log[Zn²⁺]_free_). Isotherms illustrating Zn²⁺ binding to (a) zinc finger domain ZF133 and (b) zinc clasp domain at different peptide concentrations. Relationships of the logarithmic values of domain concentration and its half-saturation points (pZn_0.5_, pZn values corresponding to 0.5 molar fractions of the formed complex) for (c) the zinc finger and (d) the zinc clasp [86].

The zinc clasp is a Zn²⁺-binding domain formed at the interface of protein molecules. Unlike classical intramolecular zinc-binding domains, the zinc clasp involves intermolecular coordination, making its assembly sensitive not only to labile Zn²⁺ concentrations but also to the concentrations of participating protein components. Examples of such intermolecular sites include the insulin hexamer, Get3 protein, the prolactin receptor in complex with growth hormone, voltage-gated potassium channels, and the MRN complex's zinc hook [23, 29, 84, 85]. The stoichiometric difference between zinc clasp and traditional Zn²⁺-protein complexes has significant thermodynamic implications. While 1:1 complexes, such as zinc fingers, show a stable fraction of Zn²⁺-bound protein at a given free Zn²⁺ concentration, zinc clasp assembly is concentration-dependent, governed by the law of mass action [86]. This is illustrated in Fig. 7: in zinc clasp formation, the pZn₀.₅ (the −log[Zn²⁺] at 50% complex formation) shifts with changing protein concentrations. Thus, zinc clasp domains are uniquely sensitive to both free Zn²⁺ and protein abundance—unlike static intramolecular zinc sites. This difference in behavior has significant implications for the functions of both types of zinc protein sites: structural and regulatory.

**Figure 8.**
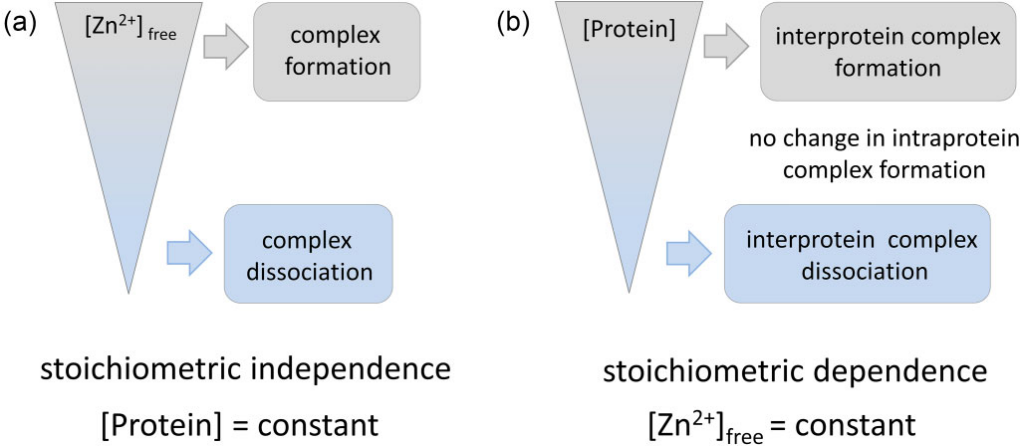
Schematic depiction of the relationship between variations in free (*in vitro*)/labile (*in cellulo*) Zn²⁺ concentrations and the assembly of protein complexes, focusing on the stoichiometry of Zn²⁺ complexes within inter- and intraprotein domains. Zn²⁺-dependent processes of complex formation and dissociation have been depicted under two conditions: (a) changes in free/labile Zn²⁺ and (b) protein subunits concentration while keeping the other variable constant [86].

The behavior of the zinc clasp domain *in vitro* provides insight into its regulatory potential *in vivo*. This is particularly well illustrated in Fig. 8. The influence of Zn^2+^ on the formation of zinc protein assemblies shows that the stoichiometry of Zn^2+^ binding sites is independent (Fig. 8a). However, the regulation of Zn^2+^ saturation among proteins is affected not only by labile Zn^2+^ concentrations but also by fluctuations in protein subunit concentrations (Fig. 8b) [86]. The ability to form intermolecular Zn²⁺ binding sites requires the physical association of two or more protein components. As protein concentrations increase, the probability of such interactions rises, leading to the formation of more ternary complexes. This principle has profound implications in the cellular context. Unlike intramolecular zinc sites that behave as static switches, zinc clasp domains offer tunable responses that reflect local cellular conditions. Importantly, this allows cells to modulate Zn²⁺-mediated protein complex formation through changes in protein abundance—without the need to disrupt global zinc homeostasis. Such a strategy is particularly advantageous when spatiotemporal separation of signaling or structural processes is required. For example, proteins may accumulate in membrane microdomains (e.g. lipid rafts or nanoclusters), where local concentration increases can drive zinc-mediated assembly, even if bulk Zn²⁺ levels remain constant. This flexible regulatory model is exemplified by the zinc clasp domain in immune cells, where CD4 is enriched in lipid rafts, likely to facilitate Zn^2+^-mediated interaction with Lck [72, 87]. While the precise contribution of Zn²⁺ in this context remains to be clarified, the dynamic spatial distribution of proteins, shaped by membrane compartmentalization and local crowding, increases the probability of intermolecular interactions and lowers the Zn²⁺ threshold needed for functional complex formation [87]. However, despite advances in proteomics, many global protein expression studies lack the spatial resolution necessary to predict such interfacial zinc assemblies [88]. Recent initiatives to map protein localization and concentration at the subcellular level show promise, but the biological variability across systems continues to challenge the precise interpretation of these data [89, 90].

In this complex environment, MTs act as the primary buffer of labile Zn²⁺ [26, 91]. These small, cysteine-rich proteins can bind up to seven Zn²⁺ ions through zinc-sulfur clusters and exist in a variety of partially saturated states Zn_7-x_MT (Zn_6_MT, Zn_5_MT or Zn_4_MT) , offering buffering from the nanomolar to the picomolar range [25]. The MT buffering capacity is often described by the T/(T + MT) ratio (thionein over total thionein + metallothionein), with a reference value of ∼0.3 across various mammalian tissues [92, 93]. The cellular Zn²⁺ pool is not a static reservoir but a dynamic system that supports and limits Zn²⁺ availability for protein complex formation. Our studies have shown that zinc clasp formation occurs within this MT buffer system, linking MT concentration and Zn²⁺ availability to the stability of the ternary protein complex [86]. Re-equilibration experiments using Lck and CD4 peptides demonstrate how both free Zn²⁺ and peptide abundance influence complex formation. This concept is supported by Rice et al., who demonstrated that fluctuations in the T/(T + MT) equilibrium directly affect the availability of labile Zn²⁺ within the cellular buffer system [94]. These changes, in turn, influence the efficiency of Zn²⁺-dependent protein interactions, such as the formation of ternary zinc clasp complexes in signaling contexts like Lck and CD4.

The functional impact of zinc sites depends not only on Zn²⁺ concentration and protein abundance but also on domain architecture and binding affinity. Structural and catalytic zinc sites, with high binding affinities, are generally saturated. In contrast, regulatory sites, such as those in MTF-1, operate at lower affinities to allow dynamic binding in response to changes in cellular Zn²⁺ [95]. Cells also regulate these sites via post-translational modifications—such as phosphorylation or S-nitrosylation of cysteine residues in Zn²⁺-thiolate clusters, which can modulate affinity and structural stability [96].

The varying affinities of the PTP family for Zn²⁺ result in different regulatory patterns that influence cellular pathways dependent on PTP activity [97, 98]. Zn²⁺ regulation is complex, involving numerous factors such as the physicochemical properties of proteins and the stability of their interactions. These factors contribute to the transient activation or deactivation of proteins. The architecture of zinc domains is crucial in determining how these parameters affect protein function, with fluctuations in labile Zn²⁺ impacting the entire zinc proteome. Cells can further enhance protein functionality by manipulating metal binding and coordination spheres, thereby introducing additional layers of regulation for functional protein complex formation.

Additionally, oxidative and nitrosative stress can modify cysteine residues in the Zn^2+^-thiolate coordination environment, which in turn affects Zn^2+^ interactions [96]. MT clusters (α and β ones) are especially susceptible to oxidation, which can lead to Zn^2+^ accumulation and neuronal cell death [99]. Moreover, redox-sensitive phosphatases can be inhibited by thiol oxidation, further linking Zn^2+^ and redox signals as integral components of cellular redox potential changes [100–102]. Through coordination, redox regulation, spatial localization, and Zn^2+^ buffering, the zinc clasp domain exemplifies the intricate regulation of interfacial Zn^2+^-mediated signaling in complex biological systems.

## Roles of CD4-Lck and CD8α-Lck coupling in T-cell immunobiology

The interaction between CD4 and CD8α co-receptors with the Src-family kinase Lck is a central mechanism regulating T-cell development, lineage stability, self-reactivity, and functional responsiveness. Recent research has shown that these interactions are not only dynamic but also distinct between CD4^+^ helper and CD8^+^ cytotoxic T cells, each contributing uniquely to T-cell biology.

In 2004, Li et al. laid the foundation for understanding the role of CD4-Lck coupling in signal amplification [103]. Their research demonstrated that in CD4-deficient T cells, Lck was not efficiently recruited, reinforcing the notion that CD4-Lck coupling is essential for full antigen sensitivity. Although they did not directly measure stoichiometry, their findings provided early mechanistic evidence that CD4 enhances TCR signaling by spatially organizing Lck at the immunological synapse. Expanding on this, Mørch et al. examined the stoichiometry of Lck binding, resolving previous uncertainties [82]. They found that CD4-Lck interactions are nearly stoichiometric, with close to 100% occupancy, whereas CD8α-Lck binding is substantial but lower, around 60%. This suggests that CD4 may have a more efficient coupling to Lck compared to CD8α, but the study did not directly explore competition between co-receptors. Notably, TCR signaling was found to induce oligomerization of the CD4-Lck complex, suggesting structural reorganization without a quantitative fluctuation in CD4-Lck binding.

Further research demonstrated that during T-cell development, the coupling between CD8α and Lck substantially increases, leading to heightened self-reactivity in peripheral CD8^+^ T cells compared to CD4^+^ T cells [104]. This difference in coupling stoichiometry appears to be an evolutionary adaptation, ensuring that CD8^+^ T cells remain primed for cytotoxic responses while CD4^+^ T cells maintain more controlled self-reactivity to support their helper functions. Further study explored this dichotomy; the role of co-receptor-bound Lck is not only quantitative but also qualitatively distinct between CD4^+^ and CD8^+^ lineages [105]. While CD8α-bound Lck is dispensable for strong antiviral and antitumor cytotoxic responses, it becomes crucial for responding to suboptimal antigens in a kinase-dependent manner. Conversely, CD4-bound Lck is indispensable for proper helper T-cell development and function, not only due to its kinase activity but also through its role in stabilizing CD4 expression at the cell surface. These findings highlight that CD4-Lck and CD8α-Lck complexes use distinct molecular mechanisms to shape their respective T-cell lineages.

The latest study added another layer of insight, revealing that Lck's association with co-receptors is crucial for maintaining lineage fidelity and antigen receptor diversity [106]. In mouse models where Lck was unable to bind to co-receptors, the antigen-specific TCR repertoires of both CD4^+^ and CD8^+^ T cells became significantly narrowed, favoring high-affinity TCRs. Moreover, this disruption led to lineage confusion, with CD4-committed cells being redirected toward the CD8^+^ lineage, especially after viral infections. These findings show the importance of Lck-to-co-receptor coupling in preserving T-cell balance and diversity, which is vital for adaptive immunity.

Recent discoveries suggest that intracellular Zn^2+^ concentrations further regulate CD4 functionality [71]. Elevated Zn^2+^ levels lead to increased partitioning of CD4 in the plasma membrane, potentially enhancing its functional availability and signaling readiness. This emerging aspect of coreceptor-Lck T-cell immunobiology presents an exciting new frontier in the research, asking for further exploration into how Zn^2+^ influences T-cell signaling and immune responsiveness.

These studies collectively demonstrate that CD4-Lck and CD8α-Lck coupling is not merely a molecular interaction but a finely tuned regulatory axis influencing T-cell development, self-reactivity, lineage commitment, repertoire diversity, and functional specialization. The evolutionary selection of distinct stoichiometries, reflected in Zn^2+^-to-proteins affinity, reflects their distinct immunological roles. In conclusion, these studies collectively illustrate that CD4-Lck and CD8α-Lck coupling is more than a simple molecular interaction. Instead, it is a finely tuned regulatory axis that influences T-cell development, self-reactivity, lineage commitment, repertoire diversity, and functional specialization. The evolutionary selection of distinct stoichiometries and functional dependencies between these two coreceptors reflects their distinct immunological roles, with CD4-Lck coupling supporting stability and helper cell function and CD8α-Lck coupling providing flexibility and responsiveness to weak antigenic signals, crucial for cytotoxic immunity.

## Biotechnological innovations

**Figure 9.**
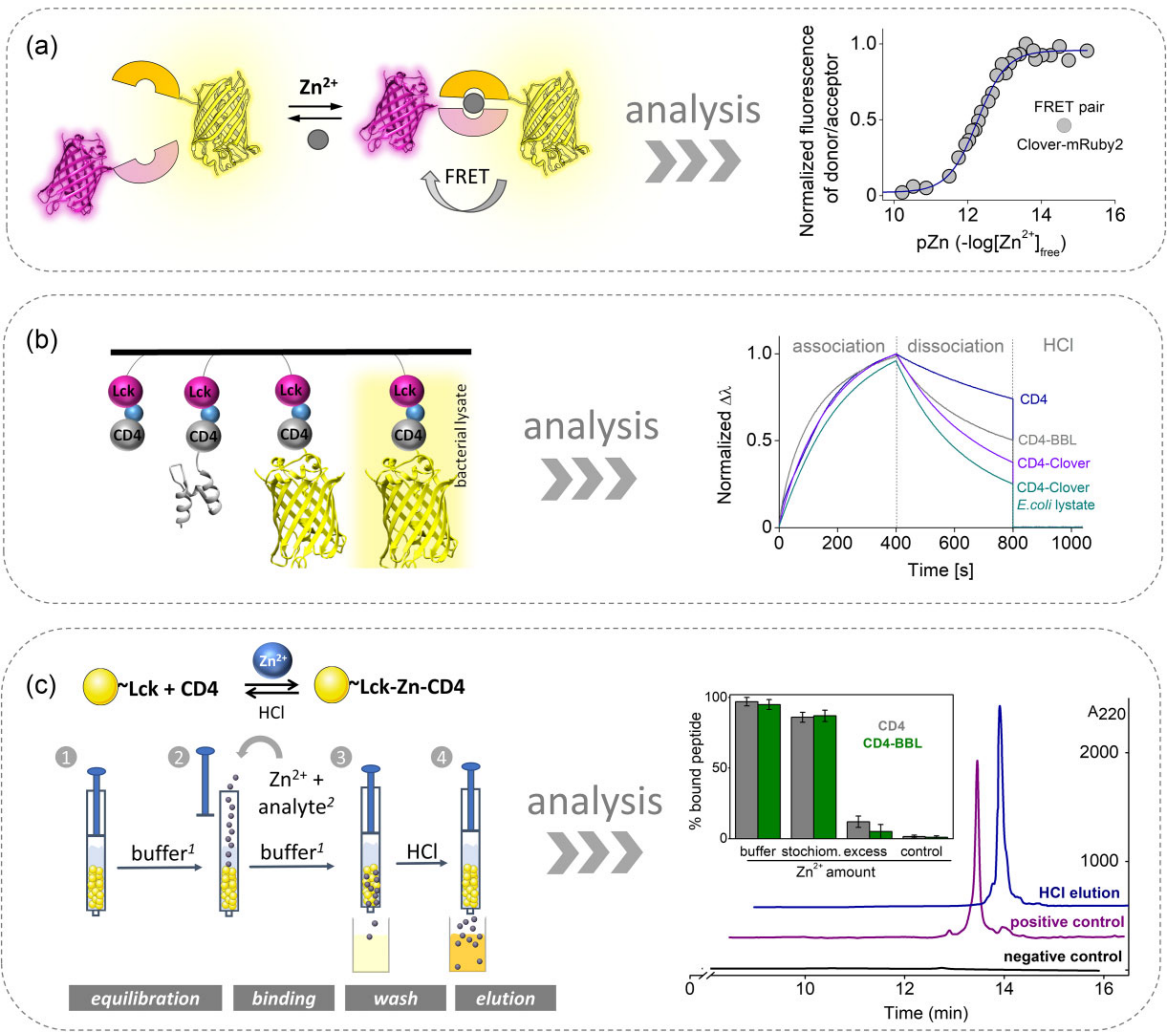
The multifunctional applications of zinc clasp tags, including the enhanced CD4RARH motif, are outlined as follows: (a) the zinc clasp tags facilitate highly efficient heterodimerization of two proteins of interest, specifically clover and mRuby2, in a Zn²⁺-dependent manner at femtomolar concentrations. The graph on the right illustrates the isotherm of zinc clasp tag assemblies as a function of free Zn²⁺ concentration in metal buffers, demonstrated through the Förster resonance energy transfer (FRET) effect. (b) The introduction of the CD4RARH tag to a protein of interest (such as BLL or clover) is achieved via native chemical ligation, followed by a Zn²⁺-dependent interaction with a biotinylated Lck motif immobilized on the surface of a streptavidin biosensor. The right graph displays sensograms depicting the Zn²⁺-dependent assembly and dissociation dynamics. The specificity of the binding interactions was also validated using an *E. coli* bacterial lysate that overexpressed the CD4RARH-Clover construct. (c) This approach also includes the use of molecular baits, specifically TentaGel™ S-NH_2_ resin coated with the Lck motif and preloaded with Zn²⁺, for the selective binding of CD4RARH and CD4RARH-tagged proteins. This method achieves ∼90% binding efficiency. The right graph shows HPLC analysis of CD4 and CD4-BBL binding to Lck-based molecular baits. Exemplary chromatograms of CD4 elution in 10 mM HCl, a positive control (unmodified resin), and a negative control (no CD4) have been presented. The inset presents a bar graph of overall results as the percentage of bound peptide regarding different Zn^2+^ conditions used.

Interprotein metal binding sites represent promising scaffolds for application in protein engineering and biotechnological endeavors [107, 108]. These sites are categorized based on their architecture into homo- and heterodimers or trimers [29, 85, 109]. A notable example of a highly stable homodimer is the zinc hook domain from the Rad50 protein, which has been effectively utilized for efficient homodimerization and as a Zn^2+^-binding motif in genetically encoded fluorescent sensors, such as the ZnGreen family and RZnP1 [110, 111]. Subsequently, leveraging this technology, a heterodimerization tool inspired by the zinc clasp domain (specifically, a variant incorporating CD4) was developed [66]. A critical aspect of this tool's development was ensuring the selectivity of the protein fragments and the overall stability of the complex, which is anticipated to be high for most biotechnological applications. To evaluate this, three different CD4 peptides (fragments 441–454, 432–454, and 421–454, Fig. 3a) were assessed for their propensity to form monomeric and dimeric species in solution. Spectroscopic and thermodynamic studies indicated that as the peptide length increased, the Zn^2+^ heterocomplex exhibited enhanced stability and selectivity for Lck. Furthermore, within the longest peptide fragment, three distinct mutations were introduced to remove potential metal-binding residues (aside from the CXC motif), specifically histidine (His) and cysteine (Cys) residues [66]. Among these mutations, only the variant with a substitution of His449 to alanine (CD4RARH) exhibited the lowest propensity for homocomplex formation while demonstrating an increased tendency for heterocomplex formation. This was evidenced by competition assays with PAR (*K*_12_ = 5.1 × 10^18^ M^−2^ at pH 7.4) and spectroscopic titrations with Co(II). The functionality, particularly the selectivity and reversibility, of the modified CD4RARH and Lck peptides was successfully validated through their fusion with fluorescent proteins (Clover and mRuby2). The assembly of these fusions was shown to be entirely dependent on pico- to femtomolar concentrations of free Zn^2+^ (Fig. 9a). This demonstrates that the incorporation of heterodimerization tags into proteins of interest (POIs) does not adversely affect the assembly and stability of the overall heterocomplex [66].

The multifunctionality of zinc clasp tags was also demonstrated through their chemical conjugation with POIs via native chemical ligation. In this context, the CDRARH peptide, which features a C-terminal dibenzyl (Dbz) moiety synthesized on a Dawson resin, was ligated to the E3-binding domain of dihydrolipoamide succinyltransferase (BBL) from *Escherichia coli*, or to the Clover protein that has an N-terminal cysteine. The binding interactions of these CD4-modified proteins with a biotinylated Lck motif immobilized on the surface of a streptavidin biosensor were investigated using biolayer interferometry (Fig. 9b). The resulting sensograms indicated that the association and dissociation of the partners were dependent on Zn^2+^ presence, exhibiting changes in response to a strong Zn^2+^ chelator or hydrochloric acid (HCl) [66]. The specificity of the binding interactions was further validated using an *E. coli* bacterial lysate that overexpressed the CD4RARH-Clover construct. In a subsequent application of the clasp-based tool, molecular baits were designed based on the Lck domain to capture CD4RARH through Zn^2+^-dependent interactions. These baits were synthesized using solid-phase peptide synthesis with Fmoc chemistry and TFA-resistant TentaGel^TM^ S-NH_2_ resin (Fig. 9c). The molecular baits, preloaded with Zn^2+^, demonstrated a remarkable capacity for selective binding of CD4RARH and CD4RARH-tagged proteins, achieving ∼90% efficiency [66]. Dissociation of the bound RARH-tagged peptide or protein occurred under slightly acidic conditions or upon the addition of an effective chelator. The successful capture of the CD4RARH and CD4RARH(BBL) conjugate by the immobilized Lck demonstrates the potential of this developed system for use in both protein heterodimerization assembly and various molecular biology applications.

An intriguing application of the zinc clasp motif in protein heterodimerization involves the identification and characterization of interactions between a fluorophore-labeled protein (designated as the “prey”) and a membrane protein (referred to as the “bait”) within live mammalian cells [112]. In this approach, cells are cultured on micropatterned surfaces that are functionalized with antibodies targeting the extracellular domain of the bait protein. The interactions between bait and prey are assessed by observing the redistribution of the fluorescently labeled prey protein. This methodology was employed to investigate the interaction between human CD4, a principal co-receptor involved in T-cell activation, and human Lck, a protein tyrosine kinase that plays a crucial role in early T-cell signaling. The authors quantified equilibrium associations by measuring the redistribution of Lck to the CD4 micropatterns and analyzed the dynamics of the interaction through photobleaching experiments and single-molecule imaging techniques. The Lck membrane anchor significantly influenced the Lck-CD4 interaction, facilitating direct binding and enhancing the stability of interactions involving other domains of Lck. Overall, the presence of the membrane anchor resulted in a two-order-of-magnitude increase in the interaction lifetime. This study highlights the importance of membrane anchoring in modulating protein-protein interactions in a cellular context.

## Conclusions and perspectives

Zinc serves as a critical modulator of T-cell biology, influencing processes ranging from early signal transduction to gene expression and functional differentiation. Its tightly regulated homeostasis, maintained by transporters such as ZIP6, ZIP8, and ZIP13, ensures precise control over intracellular Zn²⁺ concentrations during T-cell activation. These fluxes affect signaling pathways, including MAPK, NF-κB, and NFAT, while also modulating the activity of Lck kinase through inhibition of its negative regulators SHP-1 and Csk. This positions Zn²⁺ not merely as a structural ion but as a dynamic signaling mediator integral to T-cell function and fate.

A central zinc-dependent structure in T-cell signaling is the zinc clasp, an intermolecular domain coordinating CD4 or CD8α with Lck. Structural and thermodynamic studies reveal that CD4 forms a more stable complex than CD8α, reflecting differences in tail length and binding interfaces. These properties underlie distinct co-receptor roles in shaping TCR sensitivity, lineage fidelity, and effector specialization. Additionally, CD4 dimerization and palmitoylation, both influenced by Zn²⁺, enhance membrane localization and signaling robustness, underscoring the need for finely tuned control to maintain immune homeostasis. MTs further regulate the availability of Zn²⁺ for signaling by buffering its labile pool, thus modulating the formation of complexes like the zinc clasp.

Beyond immunobiology, the zinc clasp motif has been adapted as a molecular tool in protein engineering. Inspired by its selective, zinc-dependent assembly, synthetic variants have been used to guide heterodimerization and study protein interactions in live cells, offering precise control over protein complex formation under physiological Zn^2+^ conditions.

Looking ahead, deeper characterization of zinc-regulated protein assemblies at the subcellular level could unlock new avenues in synthetic biology and immunotherapy. Integrating high-resolution metal ion imaging with structural and proteomic approaches will be essential to unravel the spatiotemporal dynamics of Zn²⁺ in immune signaling. Such insights will enhance our understanding of T-cell regulation and support the development of zinc-responsive therapeutic strategies.

## Data Availability

No new data were generated or analyzed in support of this research.
